# Changes in Serbian Yellow Rust Races Reveal Genotype-Specific Responses of Yield and Quality-Related Traits in Commercial Winter Wheat

**DOI:** 10.3390/microorganisms14061217

**Published:** 2026-05-27

**Authors:** Radivoje Jevtić, Vesna Župunski, Dragan Živančev, Branka Orbović

**Affiliations:** Institute of Field and Vegetable Crops, 21000 Novi Sad, Serbia; dragan.zivancev@ifvcns.ns.ac.rs (D.Ž.); branka.orbovic@ifvcns.ns.ac.rs (B.O.)

**Keywords:** test weight, thousand kernel weight, protein content

## Abstract

Wheat yellow (stripe) rust (*Puccinia striiformis* f. sp. *tritici*) remains a major constraint to wheat production, yet relationships between infection level, yield, and quality-related traits are often inconsistent. This study evaluated how contrasting yellow rust races and infection intensities influence yield, test weight (TW), thousand kernel weight (TKW), and crude protein content in commercial winter wheat varieties. Field trials were conducted in 2016, 2021, and 2023, representing seasons that differed in yellow rust incidence and severity. The yellow rust race was changed in 2023 compared to the yellow rust race that was the same in 2016 and 2021. Associations between qualitative variables (variety and year) and quantitative variables (yield, TKW, TW, disease index (DI), and protein content) were analyzed using principal component analysis for mixed data and regression modeling. At low to moderate infection levels, TW showed a stronger negative linear association with yellow rust DI than TKW, suggesting that TW acts as an early indicator of source limitation. In contrast, TKW declined only when genotypes could no longer compensate for reduced assimilate supply, after which both traits responded similarly under severe physiological stress. Protein concentration increased under high infection levels, but its association with yield loss and DI was weak. Under high disease pressure, yield and quality-related responses were highly variable and genotype-specific at comparable DI levels, demonstrating that equivalent symptom expression does not necessarily translate into equivalent physiological disruption. These results show that yield and quality responses to yellow rust cannot be inferred from DI alone, highlighting the importance of physiological tolerance and source–sink efficiency in breeding and disease management strategies.

## 1. Introduction

Wheat is one of the world’s most significant arable crops, supplying over one-fifth of the dietary energy consumed by nearly two-thirds of the global population and accounting for roughly 30% of total grain output and about half of international grain trade [1]. Developing wheat varieties that simultaneously achieve high productivity and desirable quality remains challenging, as these traits are often weakly associated or even negatively correlated [2,3]. The development of highly adaptive wheat varieties is further complicated by the ongoing adaptation of pathogen populations under the combined selection pressures imposed by agroecological conditions, host resistance, and pesticide use [4].

*Puccinia striiformis* f. sp. *tritici*, the pathogen responsible for yellow rust, is among the most destructive diseases affecting small-grain cereals and is capable of dispersing over long distances via atmospheric currents [5,6]. Rust fungi are highly adaptive to new resistance genes and are also highly genetically diverse for races [7,8]. The adaptive potential of pathogens is strongly affected by their reproductive biology, since species with a sexual stage in their life cycle can generate higher genetic diversity, enabling faster selection of virulence and a greater ability to overcome host resistance [5,9]. Together, these interacting factors complicate effective disease management.

Until the beginning of the last decade, the *P. striiformis* f. sp. *tritici* population in Europe was considered largely clonal, with new virulent races arising mainly through mutations within existing lineages, while sexual recombination was assumed to play only a limited role [10,11,12]. A major change in the European *P. striiformis* f. sp. *tritici* race structure was observed after 2011, when new aggressive lineages, including the races known as “Warrior” and “Kranich,” were detected in several European countries and showed higher genetic diversity and broader virulence than previously dominant races [13]. These lineages spread rapidly across different parts of Europe and replaced earlier race structures, causing severe epidemics on wheat varieties that had previously been resistant [14,15]. Similar changes in race composition and disease severity have also been reported outside Europe, including in North Africa and neighboring regions, confirming the high evolutionary potential and dispersal ability of the pathogen [13,16,17]. These findings illustrate that the emergence of new *P. striiformis* f. sp. *tritici* races can quickly alter disease pressure and lead to widespread outbreaks, even in environments where resistant varieties had previously provided effective control [18].

In Serbia, changes in the predominant yellow rust races have been observed in recent years. The race detected in 2016 belonged to the Warrior lineage (PstS7), whereas isolates collected in the 2022/2023 growing season formed a distinct clade related to the PstS10 race group, indicating the emergence of a genetically different lineage in the region [19]. These findings are consistent with recent changes reported across Europe. Because the pathogen can disperse over long distances by wind, newly emerged lineages may quickly replace previously dominant races within a few seasons.

Although the effects of wheat obligate pathogens on yield are well documented, their influence on yield components and quality parameters has been less extensively studied [20]. One of the yield components closely associated with grain quality is thousand kernel weight (TKW), with higher values generally linked to improved seed vigor and favorable milling characteristics [21]. Test weight (TW) is widely employed as a key commercial standard in wheat trading. However, its reliability as a predictor of milling performance is variable and depends on wheat class, genetic differences among varieties within a class, and the environmental conditions during crop production [22]. Crude protein content is commonly used as a minimum quality criterion and is known to be strongly influenced by climatic conditions and agronomic practices [23,24].

Although yield and quality-related parameters are known to be affected by abiotic factors, disease pressure, and genotype, their combined effects are still insufficiently understood [25,26]. Jevtić et al. [27] demonstrated that genotype-specific responses to climatic factors at particular phenological stages can substantially influence interactions among obligate pathogens and determine their relative dominance. Although infection level and its effects on yield and quality are generally more pronounced in highly susceptible and susceptible varieties, previous studies have also shown that disease index and yield loss caused by obligate pathogens are not always strongly correlated, even in susceptible genotypes [4]. In addition to this, more recent research, has also emphasized the importance of the combined effects of abiotic and biotic stressors on wheat performance [28,29].

Differences in the effects of yellow rust races remain insufficiently explored, particularly regarding yield, while relationships between yield and quality-related traits are complex and environmentally driven. It was therefore hypothesized that the impact of yellow rust on quality-related traits in winter wheat cannot be explained solely by climatic effects on the pathogen life cycle or resistance gene effectiveness, but rather reflects the overall genetic capacity of varieties to cope with combined abiotic and biotic stresses.

Accordingly, the objectives of this study were: (1) to evaluate how different yellow rust races and levels of infection influence quality-related traits—namely thousand kernel weight (TKW), test weight (TW), and grain protein content—in the set of commercial winter wheat varieties, and (2) to assess the relationships between these quality parameters and yield under exposure to diverse yellow rust races. Because the effects of yellow rust on quality-related traits have been studied far less than its effects on yield, this work aims to provide broader insight into how shifts in yellow rust races influence the overall commercial performance of winter wheat.

## 2. Materials and Methods

### 2.1. Wheat Field Trials

Field experiments were conducted during the 2016, 2021, and 2023 growing seasons at the Rimski Šančevi experimental station (45°19′28″ N, 19°50′25″ E; altitude 80.24 m), located in Vojvodina, northern Serbia. The trials were designed to evaluate the resistance and susceptibility of commercial winter wheat varieties to *P. striiformis* f. sp. *tritici*. Experiments were established on a slightly calcareous loamy chernozem soil. The trials were conducted following the methodology recommended by CIMMYT [30]. Each genotype was sown so that the total row length per genotype was equivalent to 5 m.

Climatic conditions were more favorable for yellow rust infection in 2016 and 2023 than in 2021 at the experimental site, with different pathogen races occurring in 2016 and 2023, while a corresponding shift in race composition was also observed at the regional scale [19]. In Serbia, the yellow rust race detected in 2016 was Warrior (PstS7 lineage), whereas isolates collected in the 2022/2023 growing season formed a distinct clade related to the PstS10 race group, indicating the emergence of a genetically different lineage that has not yet been formally designated as a race [19]. In 2021, climatic conditions were not conducive to yellow rust infection and were also restrictive for wheat growth due to lower total precipitation than the eighteen-year average during the flowering and grain-filling period (Table 1). Monthly mean air temperature and total precipitation for the three growing seasons (2016, 2021, and 2023) were obtained for the experimental location from the meteorological station at Rimski Šančevi operated by the Republic Hydrometeorological Institute of Serbia (RHMZ; http://www.hidmet.gov.rs/).

In total, 11 commercial varieties were evaluated in 2016, 2021, and 2023 for yellow rust disease index (DI), test weight (TW), thousand kernel weight (TKW), and yield. The varieties were commonly grown in Serbia and represent genotypes adapted to the agroecological conditions of the Pannonian region, differing in agronomic performance and disease response. The names of the varieties are not reported to avoid potential conflicts of interest and to focus on general response patterns rather than on the performance of individual commercial varieties.

Crude protein content was evaluated 40 days after harvest in 35 commercial varieties in 2021 and 2023 under conditions with the presence and absence of yellow rust infection in the same year. To compare crude protein performance under conditions with and without yellow rust infection, each variety established in plots covering 5 m^2^ was subdivided into fungicide-treated (1 m^2^) and untreated (4 m^2^) sections. Fungicide applications were carried out at the recommended rates at growth stage BBCH 65. In 2021, treatments contained prothioconazole (100 g L^−1^; 10.3% *w*/*w*), tebuconazole (100 g L^−1^; 10.5% *w*/*w*), and spiroxamine (250 g L^−1^; 26.3% *w*/*w*), whereas in 2023 benzovindiflupyr (75 g L^−1^) and prothioconazole (150 g L^−1^) were applied. Applications were made using calibrated field sprayers equipped with flat-fan nozzles, operating at a pressure of 300 kPa and delivering 200 L ha^−1^ of spray solution. Fungicide-treated plots were included as a disease-controlled reference to enable the estimation of grain yield loss and crude protein content increment, as defined in the subsequent section. Winter wheat was sown at the optimal planting time, with an average sowing date of 20 October, and harvested at physiological maturity, with a mean harvest date of 30 June.

### 2.2. Trait Assessments

#### 2.2.1. Disease Index

The severity of obligate fungal diseases was evaluated at growth stages BBCH 71–73 (watery ripe to early milk), which are closely associated with yield determination [31]. These stages were selected because grain filling occurs during this period and key yield and quality-related traits, including grain weight, test weight, and protein content, are largely determined at this time, while foliar diseases affecting photosynthesis can strongly influence assimilate supply to the grain [20].

Disease severity was scored using a modified Cobb scale [32,33], a widely used method for visual estimation of rust severity in field experiments, including studies on stripe rust [34]. The disease index (DI) for yellow rust was calculated by integrating disease incidence with mean severity and expressed as a percentage [35].

#### 2.2.2. Grain Yield

Grain yield was determined for each plot and standardized to a moisture content of 15%. Yield loss was calculated as the percentage reduction in yield in untreated plots relative to the corresponding fungicide-treated plots, which represented conditions with effective disease control. Yield loss was calculated according to Equation (1), where:

(1) $$Y\left(\%\right)=((Y_{1}-Y_{2})/Y_{1})\times 100$$

- *Y*_1_ = grain yield of the fungicide-treated plot representing optimal disease control.
- *Y*_2_ = grain yield of the non-sprayed (untreated) control plot.

#### 2.2.3. Crude Protein Content

Crude protein content was quantified using a handheld GrainSense near-infrared (NIR) analyzer (GrainSense Ltd., Oulu, Finland) and expressed on a dry matter basis. The accuracy of protein measurements obtained with this device has been previously validated against the standard Kjeldahl method, showing a strong positive correlation (r = 0.951) [36]. Measurements were completed within approximately 3 s using samples of 60–80 kernels. The analyzer operates using NIR spectroscopy with full-sample illumination provided by an integrating sphere, ensuring uniform light penetration. Crude protein content increment was calculated according to Equation (2), where:

(2) $$Y\left(\%\right)=((Y_{1}-Y_{2})/Y_{2})\times 100$$

- *Y*_1_ = protein content of the fungicide-treated plot representing optimal disease control.
- *Y*_2_ = protein content of the non-sprayed (untreated) control plot.

In contrast to grain yield, which is expressed as loss, crude protein content was evaluated as an increment, as it generally increased under yellow rust infection. This reflects the compositional nature of protein concentration, which depends on the balance between nitrogen accumulation and carbohydrate deposition in the grain.

### 2.3. Statistical Analysis

The effects of variety, year, the variety × year interaction, and disease index on yield, thousand kernel weight (TKW), test weight (TW), and crude protein content were evaluated using multiple regression analysis. Additional regression models were used to identify the main factors influencing yield loss and crude protein increment in fungicide-untreated plots.

In all regression analyses, disease index was included as a continuous predictor, while variety and year were treated as categorical variables. In the regression models, the disease index (DI) was included as a continuous covariate describing infection intensity rather than as an experimental factor, allowing separation of the effects of infection intensity from the effects of year and variety. Stepwise multiple regression was used for variable selection in models including several abiotic and biotic predictors potentially associated with yield and quality-related traits. Because these predictors may also be intercorrelated, multicollinearity was evaluated using variance inflation factors (VIF). The entry and removal criteria in the stepwise procedure were set at α = 0.15, as recommended when biologically relevant variables may otherwise be excluded from the model [37].

Associations between categorical variables (variety and year) and quantitative variables (yield, TKW, TW, and disease index) were further explored using principal component analysis for mixed data (PCAmix), which integrates principal component analysis (PCA) and multiple correspondence analysis (MCA) [38]. PCAmix was also used to visualize the associations of yellow rust with crude protein content, yield loss, crude protein increment, and year.

To compare mean values and quantify differences among treatments, a general linear model followed by Tukey’s post hoc test was applied at a 95% confidence level. Relationships among variables were assessed using Spearman’s rank correlation coefficient. All statistical analyses were performed using Minitab Statistical Software (version 17.1.0) and XLSTAT (trial versions), with graphical visualization of PCAmix results conducted in R using the *ggplot2* package.

## 3. Results

### 3.1. Contrasting Yellow Rust Race Effects on Disease Occurrence and Genotype-Specific Yield and Quality-Related Responses in Commercial Wheat

**Figure 1:**
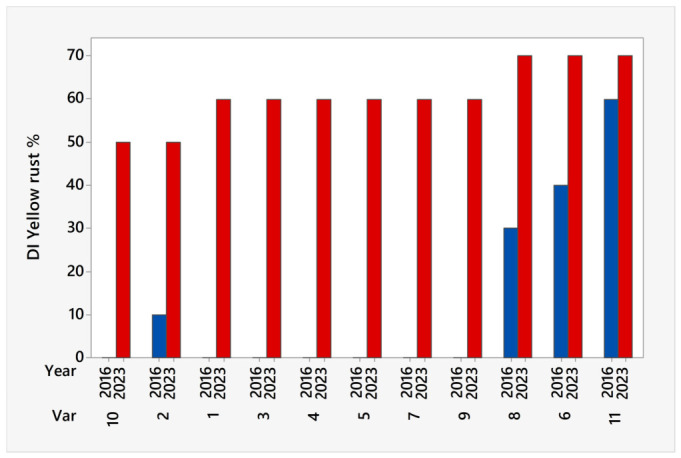
Yellow rust DI was substantially higher in the 2023 growing season than in 2016. Seven of the eleven varieties that exhibited only trace levels of yellow rust infection in 2016 became highly susceptible in 2023 at the Rimski Šančevi locality. A total of 11 commercial winter wheat varieties were assessed for yellow rust severity and incidence in both 2016 and 2023 at growth stages BBCH 71–73 (watery ripe to early milk), which are closely associated with yield determination. Disease indices (%) were calculated by integrating disease incidence and average disease severity.

To assess the reaction of commercial varieties to yellow rust infection and to determine whether the effects of yellow rust on yield and quality-related traits of winter wheat have changed over time, the disease indices were analyzed in 2016 and 2023, both years favorable for infection. A previous study [19] indicated that the yellow rust race in Serbia changed in 2023. In this study, it was found that *P. striiformis* f. sp. *tritici* infection increased significantly in 2023 (DI__AVER_ = 60.8%) compared with 2016 (DI__AVER_ = 11.8%). All tested commercial varieties were infected in 2023, with yellow rust DI values exceeding 50%, whereas in 2016, 7 out of 11 varieties showed only trace levels of yellow rust infection (Figure 1). A greater aggressiveness of *P. striiformis* f. sp. *tritici* in 2023 was also indicated by the higher percentage of varieties with a DI of 60% in 2023 (54.5%) compared with 2016 (9.1%).

**Figure 2:**
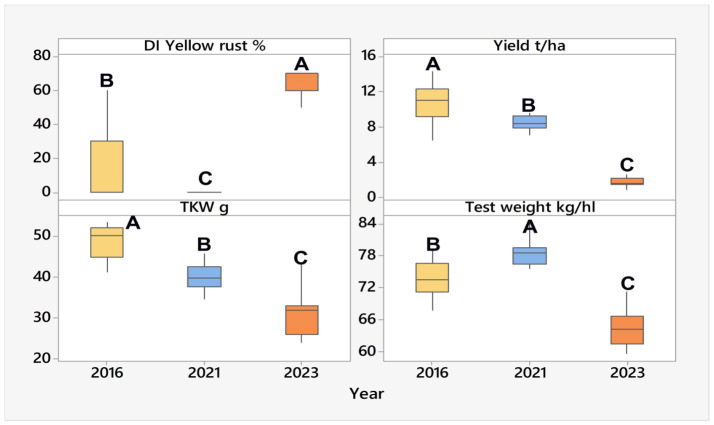
Yield, thousand kernel weight (TKW), and test weight (TW) of 11 commercial winter wheat varieties evaluated in 2016, 2021, and 2023. The seasons differed in yellow rust occurrence, with Warrior (PstS7) detected in 2016 and a distinct lineage related to PstS10 detected in 2023. Tukey’s pairwise comparisons (95% confidence interval) were used to test differences among years for disease index (DI), yield, TKW, and TW. Means sharing the same letter are not significantly different at α = 0.05.

A significant effect of year on yellow rust infection (*p* < 0.001) was confirmed by ANOVA and Tukey’s pairwise comparisons at 95% confidence (Figure 2). Although 2021 was not favorable for yellow rust infection, it was included in the analysis of yield and quality-related traits to evaluate varietal responses to abiotic stress in the absence of disease.

On average, yield (1.7 t ha^−1^), thousand kernel weight (TKW; 30.6 g), and test weight (TW; 64.4 kg hL^−1^) were significantly lower in the season characterized by the emergence of a new yellow rust race (2023) than in 2016, when the previously prevailing, less aggressive yellow rust race dominated and yield, TKW, and TW reached 10.8 t ha^−1^, 49.1 g, and 73.6 kg hL^−1^, respectively. Moreover, in the year without yellow rust infection (2021), yield (8.3 t ha^−1^) and TKW (39.9 g) exceeded those recorded in 2023, but remained significantly lower than in 2016. In contrast, test weight in 2021 (78.5 kg hL^−1^) was significantly higher than in both 2016 and 2023, which were years favorable for yellow rust infection (Figure 2; Table 2).

**Figure 3:**
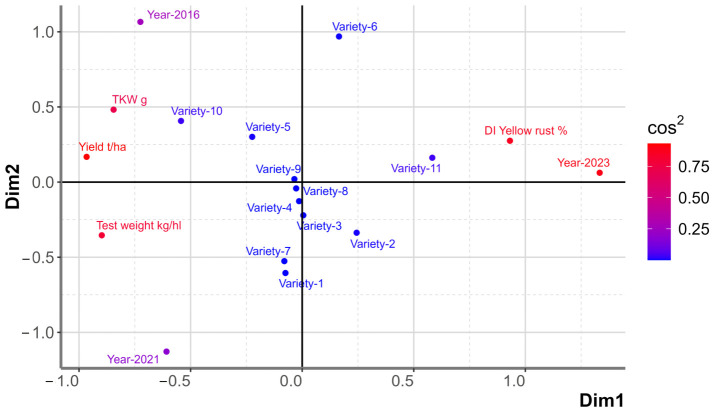
PCAmix analysis showing negative associations between yellow rust disease index (DI) and thousand kernel weight (TKW), test weight (TW), and yield among 11 commercial winter wheat varieties evaluated in 2016, 2021, and 2023 at the Rimski Šančevi locality. The biplot illustrates relationships among yield, TKW, TW, yellow rust disease index, years, and varieties across the three growing seasons.

To visualize the global pattern within the data and the associations of yellow rust with yield, year, and quality-related traits, PCAmix was performed. The first two principal components explained 35.85% of the overall variability. The contribution of the first and second dimensions was 26.7% and 9.1%, respectively (Figure 3). DI of yellow rust was positioned on the opposite side of the biplot origin relative to yield, TKW, and TW, indicating a negative association between disease index and these traits. In addition, DI was also more associated with 2023 when a new race occurred.

PC1 represented the main disease–performance gradient, with yellow rust disease index (DI) and the year 2023 loading positively, while grain yield, thousand kernel weight (TKW), and test weight (TW) loaded negatively, indicating a strong negative association between disease index and agronomic performance. PC2 separated yield and TKW (positive loadings) from TW (negative loading), reflecting differences in grain-filling responses among varieties. Varieties more closely associated with TW clustered with the year 2021, which was characterized by the absence of yellow rust infection but by restricted growing conditions due to drought during anthesis and grain-filling. The dispersion of varieties across all quadrants of the biplot indicates substantial heterogeneity in varietal responses to both disease pressure and grain-filling conditions, contributing to the relatively moderate proportion of variability explained by the first two components.

Since yield, TKW, and TW showed distinct associations with yellow rust disease index (DI) among varieties, their relationships were further analyzed in two years characterized by different yellow rust races (2016 and 2023).

**Figure 4:**
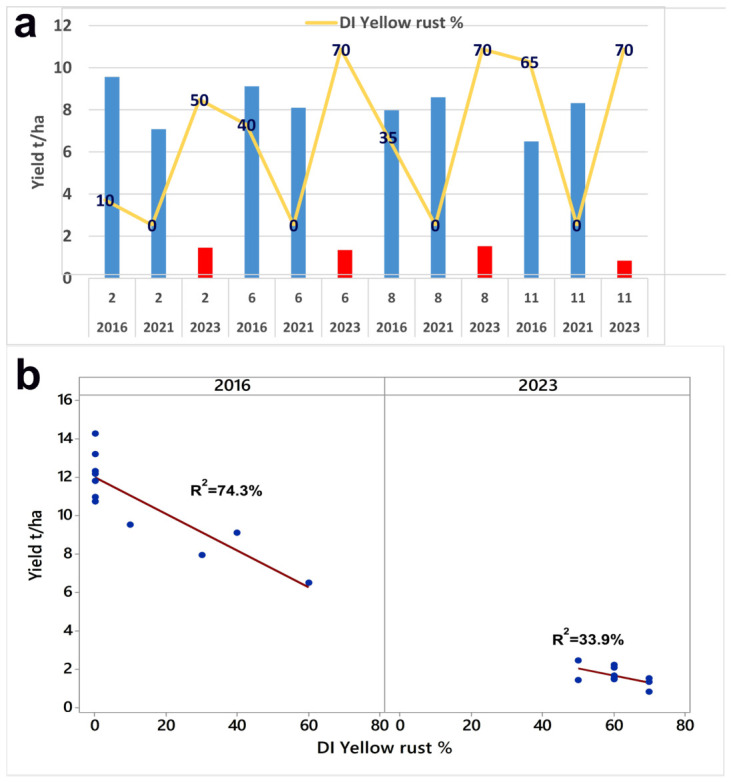
The relationship between yield response and yellow rust infection level was not straightforward. (**a**) For four commercial winter wheat varieties evaluated at the Rimski Šančevi locality, similar yield levels were observed across contrasting yellow rust disease index (DI) values in 2016 and 2023, suggesting that yield did not change proportionally with DI. (**b**) Regression analysis was used to evaluate the relationship between yield and DI, which was stronger at low to moderate infection levels than at high infection levels, where responses became more variable among varieties.

This study showed that the relationship between DI of yellow rust and yield response was not straightforward. This result showed that susceptible genotypes can give yields over 9 t/ha although infected with different DIs such as DI 10% (Variety 2), and DI 40% (Variety 6), as was the case in 2016. However, DIs over 40% in 2023 were associated with much lower yields, and yields did not exceed 2 t/ha in any variety. In addition, high yellow rust infection was not always associated with yield reduction at the same DI level. For example, Variety 11 infected with 65% DI in 2016 reached a yield of 6.5 t/ha, whereas in 2023 its yield was below 2 t/ha at 70% DI (Figure 4a).

Overall, under low to moderate infection levels in 2016, an R^2^ of 74.3% indicated a strong linear relationship between yellow rust infection level and yield (Figure 4b). However, under high infection levels, the effect of yellow rust on yield became more variable and genotype-specific, even at similar DI values, resulting in a lower coefficient of determination (R^2^ = 33.9%) (Figure 4b).

**Figure 5:**
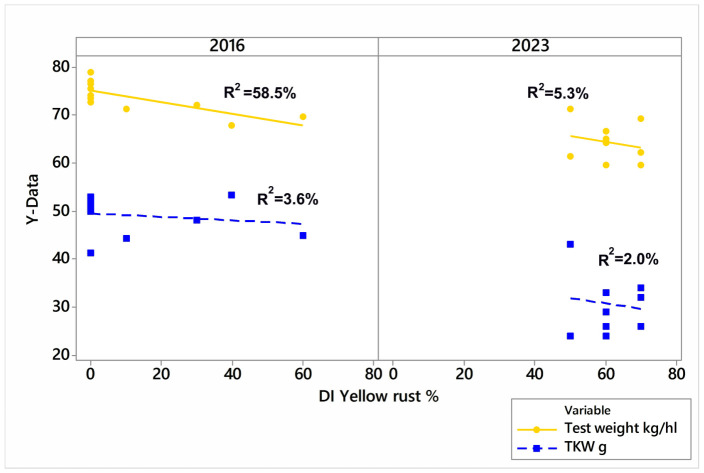
The association between yellow rust disease index (DI) and test weight (TW) of 11 commercial wheat varieties was stronger in 2016, under low to moderate infection levels, than in 2023, when a different yellow rust race predominated. Under high infection levels, responses among varieties became more variable, reducing the strength of the association. In contrast, the relationship between thousand kernel weight (TKW) and yellow rust DI remained weak in both years.

Regression modelling of the relationships between yellow rust disease index (DI) and quality traits in 2016 and 2023—years with different predominant yellow rust races—showed that at low to moderate infection levels in 2016, test weight (TW) exhibited a stronger linear association with DI (R^2^ = 58.5%) than did thousand kernel weight (TKW) (R^2^ = 3.6%) (Figure 5). In contrast, under high infection levels in 2023, coefficients of determination for TW (R^2^ = 5.3%) and TKW (R^2^ = 2.0%) were low, indicating greater variability of responses among varieties at similar DI values (Figure 5). When both years were analyzed together, the correlation between TW and DI was significant and strongly negative (r = −0.847, *p* < 0.001), as was the correlation between TKW and DI (r = −0.737, *p* < 0.001) (Table 3). These results indicate that increasing yellow rust infection was associated with reductions in both TW and TKW, but differences among genotypes were more pronounced under high infection levels.

**Figure 6:**
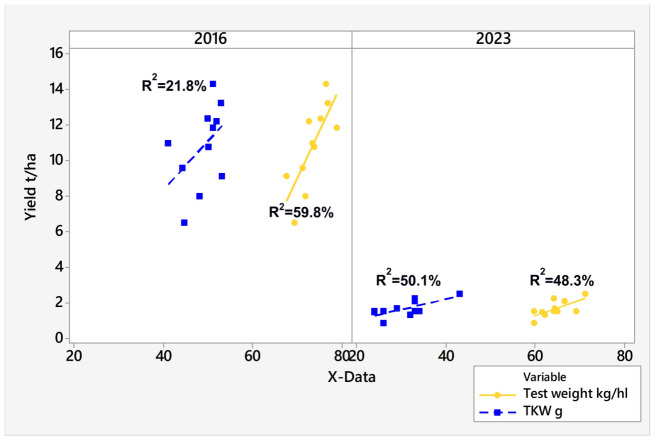
Regression modeling showed a stronger association between yield and test weight (TW) than between yield and thousand kernel weight (TKW) of 11 commercial varieties under low to moderate infection levels in 2016. In contrast, under high disease pressure, the relationships of both TKW and TW with yield were similar, showing moderate levels of association.

Given that yield and quality-related traits are controlled by different, but interrelated mechanisms, regression analysis was performed to examine how changes in yield were associated with changes in quality-related traits (TW and TKW) in years characterized by different yellow rust races (2016 and 2023). In 2016, when yields exceeded 5 t/ha, TW exhibited a more linear relationship with yield (R^2^ = 59.8%) than TKW (R^2^ = 21.8%) (Figure 6). Under high infection levels in 2023, TW and TKW showed similar associations with yield, with R^2^ values of 48.3% and 50.1%, respectively.

**Figure 7:**
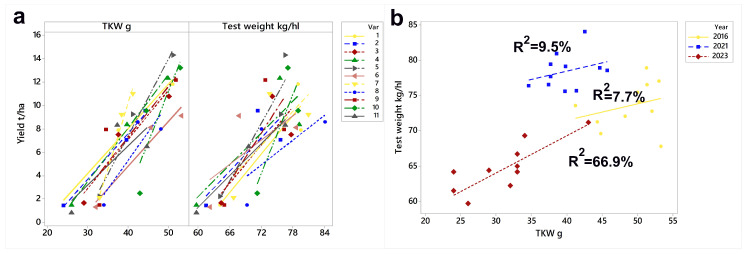
Effects of variety on the relationships between yield and quality-related traits (thousand kernel weight (TKW) and test weight (TW)), and the influence of contrasting pathogen pressure among years on the relationship between TKW and TW for 11 commercial winter wheat varieties grown at the Rimski Šančevi locality in three seasons (2016, 2021, and 2023). (**a**) The relationships of TKW and TW with yield were strongly variety-dependent, reflecting differences in varieties capacity to cope with varying environmental conditions. (**b**) Under high yellow rust infection pressure (2023), the association between TKW and TW was stronger than in years with low to moderate infection (2016) or in the absence of infection (2021).

Differences among varieties in the regulation of the relationship between yield and quality-related traits were also demonstrated by linear regression analyses that included all three years, with and without yellow rust infection. The coefficient of determination (R^2^) ranged from 46.0% to 87.2% for associations between yield and test weight (Figure 7a) for 11 varieties. The same was true for the relationship between TKW and yield in 11 varieties with the coefficient of determination (R^2^) ranging from 64.6% to 98.3% (Figure 7a). The association between test weight (TW) and thousand kernel weight (TKW) differed among seasons, showing a weak association in the year with a less aggressive yellow rust race (2016; R^2^ = 7.7%) and in the year with no yellow rust infection, but unfavorable for wheat growth (2021; R^2^ = 9.5%). In contrast, in 2023, when a more aggressive race predominated, the association between TW and TKW was much stronger (R^2^ = 66.9%) (Figure 7b).

### 3.2. High Yellow Rust Infection Elevates Protein Content Without Strong Yield Correlation

Because grain protein concentration is strongly influenced by environment and yield-driven dilution effects, protein responses were evaluated using a contrasting-year approach (severe infection vs. no infection), recognizing that this comparison captures the combined influence of yellow rust presence and year-specific growing conditions rather than a pure race effect.

To visualize the global pattern within the data and the associations of yellow rust with crude protein content, yield loss, crude protein increment, and year, PCAmix was performed. The first two principal components explained 82.96% of the overall variability. The contribution of the first and second dimensions was 66.5% and 16.4%, respectively.

**Figure 8:**
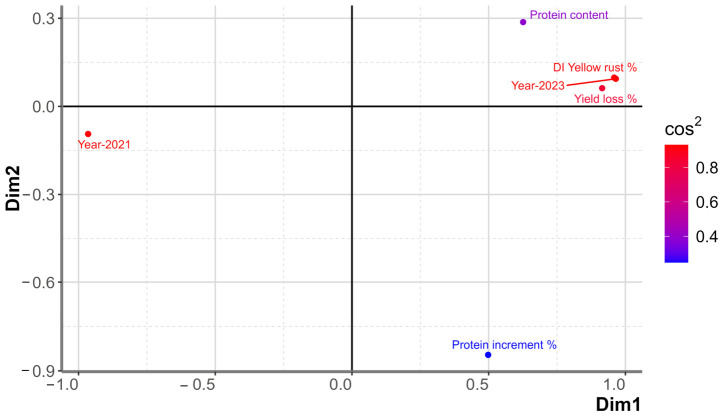
PCAmix analysis showing close associations between yellow rust disease index (DI) and yield loss among 35 commercial winter wheat varieties evaluated in 2021 and 2023 at the Rimski Šančevi locality. The biplot illustrates relationships among protein content, yield loss, protein content increment, yellow rust disease index, and years across the two growing seasons.

In the PCA biplot, the years 2021 and 2023 were positioned on opposite sides of the origin, reflecting contrasting disease environments. Yellow rust disease index (DI) and yield loss were more closely associated than DI with protein content. In contrast, protein increment (%) was located in the lower-right quadrant and loaded negatively on PC2, suggesting that the magnitude of protein increase depended not only on disease severity but also on genotype-specific baseline protein levels and other contributing factors (Figure 8).

**Figure 9:**
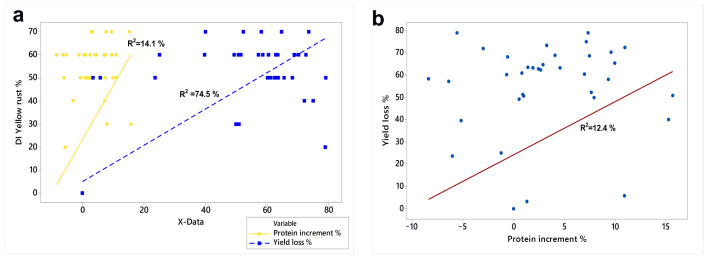
Regression analysis used to evaluate relationships between (**a**) yellow rust disease index (DI), protein increment, and yield loss, and (**b**) yield loss and crude protein increment for 35 commercial wheat varieties evaluated in two years (2021 and 2023) at the Rimski Šančevi locality. The results showed a stronger association between DI and yield loss than between DI and protein increment, whereas the relationship between protein increment and yield loss was more variable among varieties.

Regression modeling confirmed the patterns observed in the PCAmix analysis. The relationship between yield loss and yellow rust disease index (DI) was stronger (R^2^ = 74.5%) than the association between DI and protein content increment (R^2^ = 14.1%) (Figure 9a). Moreover, the relationship between yield loss and protein content increment was relatively weak (R^2^ = 12.3%) (Figure 9b). This indicates that the increase in protein content was not directly proportional to yield reduction. Although an inverse relationship between yield and protein concentration is commonly observed, the present results show that the associations of DI with yield loss and protein response differed in magnitude. This indicates that the increase in protein content was not simply a consequence of yield reduction, suggesting that the relationship between yield loss and protein response varied among varieties and years. These results are consistent with the PCA, in which protein increment and protein content loaded on opposite sides of PC2, separating disease-driven protein response from genotype-dependent protein levels.

## 4. Discussion

This study showed that shifts in yellow rust races were associated with significant reductions in yield, TKW, and TW, and with an increase in crude protein content. However, the results also indicated that there was no consistent relationship between disease index (DI) and the magnitude of impairment of yield or quality-related traits. Similarly, the associations between DI and test weight (TW), thousand kernel weight (TKW), and crude protein content did not depend on infection intensity alone, but also on genotype-specific physiological responses of the host plant under disease pressure. Because the study was conducted under naturally occurring field conditions, the observed differences among years should be interpreted as the combined effect of pathogen race background, infection intensity, year-specific environmental conditions, and genotype-specific responses, rather than as the result of a single causal factor.

### 4.1. Yield and Quality-Related Responses of Commercial Wheat Varieties Under Contrasting Yellow Rust Races

The occurrence of yellow rust outbreaks is primarily associated with the emergence of new virulent races capable of overcoming resistance genes and with the widespread cultivation of susceptible varieties, while climatic conditions during the growing season influence the development and severity of epidemics [39,40,41,42,43,44,45,46]. In agreement with this, the present study suggests that the magnitude of yield loss was influenced not only by the level of yellow rust infection, but also by the prevailing yellow rust race, year-specific environmental conditions, and genotype-specific physiological mechanisms governing plant defense and stress tolerance. In 2016, favorable for wheat growth, different disease index (DI) levels were associated with similar yield performance across varieties. Moreover, susceptible varieties achieved higher yields in a season conducive to yellow rust development (2016) than in a year unfavorable for yellow rust, but also with limiting wheat production conditions (2021). Interestingly, the high level of yellow rust infection on the same variety in two years with different predominant yellow rust races (2016 and 2023), showed significantly different yield responses.

The shift in yellow rust races in 2023 was documented by Župunski et al. [19]. In this study, the change in the predominant yellow rust race coincided with significant reductions not only in yield but also in key quality-related traits. In addition, this study allowed assessment of the relationships between quality-related traits and yellow rust disease index across the full range of infection levels, from low to high. Thousand kernel weight (TKW) exhibited a weaker association with yellow rust disease index (DI) than test weight under low to moderate infection. This suggests that these two traits were influenced through partly different physiological processes. Yellow rust, a biotrophic pathogen, reduces the photosynthetic activity of infected leaves and accelerates their senescence, which in turn limits the carbohydrate supply required for grain filling [47]. Test weight reflects grain density and uniformity and is therefore highly responsive to disruptions in grain filling. In contrast, TKW mainly reflects kernel-level growth and sink strength, which depend on genotype-specific regulation and reserve remobilization capacity [48,49,50]. Consequently, under low to moderate disease pressure, TW responded more directly to reductions in source activity, whereas TKW appeared more buffered. This is also confirmed by the weak relationship between TKW and TW in the years with less aggressive yellow rust and in the drought year without yellow rust infection, indicating that TKW and TW were regulated by largely independent, genotype-specific mechanisms.

At higher infection levels, trait–DI relationships became increasingly variable among varieties, indicating that yield and quality expression were governed more by differences in host tolerance and physiological adjustment capacity. A strong relationship between TKW and TW was observed only in the year with a more aggressive yellow rust race, suggesting that under severe disease pressure, both traits were influenced by similar physiological constraints across genotypes.

The observed pattern suggests the existence of genotype-specific thresholds in the response of grain traits to yellow rust stress. Under low to moderate infection levels, test weight was more closely associated with source disruption than TKW, reflecting its sensitivity to changes in grain uniformity, endosperm packing, and the proportion of partially filled kernels. In contrast, many genotypes were still able to buffer individual kernel mass through reserve remobilization and adjustments in grain filling, resulting in a weaker response of TKW at these infection levels. When infection intensity increased in the presence of the more aggressive race, the compensatory capacity of the host was eventually exceeded. Grain filling became uniformly source-limited, leading to parallel declines in both TKW and TW and to a strong inter-trait correlation. These results indicate that infection level alone does not determine trait responses; rather, genotype-specific tolerance defines the threshold at which distinct quality traits converge onto a common physiological limitation. Because these observations were obtained under field conditions without controlled inoculation, the proposed physiological interpretation should be considered a plausible explanation supported by the observed patterns and literature, rather than as direct experimental proof of the underlying mechanisms.

Similar observations were reported by Župunski et al. [19], who found that yield losses among wheat varieties varied independently of yellow rust infection levels. Previous studies on other wheat pathogens have likewise shown that the impact of fungal diseases on wheat performance is not always closely correlated with the level of infection. Jevtić et al. [51] demonstrated that individual genotypes can tolerate defined levels of *Fusarium* head blight (FHB) pressure without a measurable reduction in yield potential. Consistently with this, Pan et al. [52] reported that defense responses to FHB are genotype-dependent, with resistant lines displaying distinct transcriptomic patterns during *Fusarium*–host interactions. Comparable variability has also been observed for yellow rust: Jevtić et al. [27] found that susceptible varieties responded differently across seasons favorable for infection. Similarly, Zhou et al. [53] showed that a yellow rust–susceptible variety (MM367) outperformed a resistant variety (XY6) in yield under both fungicide-treated and untreated conditions. Given that the regulatory networks governing plant responses to abiotic and biotic stresses comprise multiple interacting components that may act synergistically or antagonistically [54,55,56], the results of this study indicate that further investigation is needed to clarify how integrated plant defense responses contribute to final yield and quality-related traits under yellow rust infection, including the possible influence of yellow rust on plant responses to other pathogens that may become epidemic later in the growing season, such as brown rust or *Septoria*, rather than focusing solely on individual resistance genes or isolated mechanistic pathways. Future studies including controlled inoculation with defined races and multi-environment testing will be necessary to separate the relative contributions of pathogen genotype, environment, and host physiology to the observed responses.

This study also indicated differences among varieties in the relationships between yield and quality-related traits. Differences among varieties were also evident in the relationships between yield and quality-related traits, indicating that these associations were strongly genotype-dependent. Similarly, Jevtić et al. [4] reported that the relationship between yield loss and TKW loss differed among wheat genotypes, which is in agreement with the findings of Lopes et al. [57], who demonstrated that TKW is strongly influenced by both varietal characteristics and environmental conditions. More broadly, reported differences in the strength and even the direction of yield–TKW relationships across studies highlight that these traits are highly dependent on variety properties, cultivation practices, and environmental factors. This suggests that reductions in yield may in some cases be accompanied by changes in TKW, but such trade-offs appear to be strongly genotype- and environment-specific.

### 4.2. Protein Content Response to High Yellow Rust Infection and Its Weak Relationship with Yield

Comparisons between years differing in infection level and predominant yellow rust race were most informative for TW and TKW, which are tightly linked to grain-filling physiology and canopy source limitation—processes that may vary with epidemic timing and race aggressiveness. In contrast, crude protein concentration is highly sensitive to seasonal conditions and yield–protein dilution [58]. Therefore, protein responses were assessed using a contrasting-year design (severe infection vs. no infection), acknowledging that this captures the integrated effect of yellow rust under differing environments.

In this study, protein content increased under high levels of yellow rust infection, yet its association with yield loss remained weak, whereas yield loss was strongly related to disease index (DI). The relationship between yield loss and DI was much stronger than the association between DI and protein increment. This divergence reflects the fundamentally different physiological bases of yield and protein responses to disease.

Grain protein concentration reflects the balance between nitrogen accumulation and carbohydrate deposition, and is therefore governed largely by yield-driven dilution [59,60]. Under yellow rust infection, reduced canopy photosynthesis and shortened green leaf duration have been reported to limit carbohydrate accumulation in developing grains, while nitrogen remobilization from vegetative tissues may be less proportionally affected, since starch and protein deposition in the grain appears to be controlled by separate mechanisms [60]. Moreover, nitrogen remobilization from vegetative tissues to the grain often continues under yellow rust infection, and its transfer to the grain varies among genotypes and environments. Consequently, infection level may determine the direction of protein change, while its magnitude is governed by source–sink adjustment capacity, explaining the weaker association between protein content and disease index than between yield and disease. In this study, the relationship between yield loss and protein content increment was rather weak, indicating that yellow rust affected both traits but with different magnitudes.

The weak coupling between protein increment and DI observed here supports the observation that yellow rust infection level is a strong predictor of yield loss, while protein concentration reflects compositional adjustment to reduced carbohydrate deposition and also indicates why in this study protein behaved differently from TW and TKW.

## 5. Conclusions

This study demonstrates that yield and quality-related trait responses to yellow rust are not determined by infection level alone, particularly under high infection pressure. At comparable disease indices, yield, test weight (TW), thousand kernel weight (TKW), and protein content showed substantial genotype-specific variation, indicating that equivalent symptom expression does not necessarily translate into equivalent physiological disruption.

- Under high infection levels, the effects of yellow rust on yield and quality-related traits became more variable and genotype-specific, even at similar disease index values.
- Under low to moderate infection levels, TW responded more directly to yellow rust DI than TKW, suggesting that TW acts as an early indicator of source limitation.
- In contrast, TKW declined only when genotype-specific compensation capacity was exceeded, after which both traits responded in parallel under severe physiological constraint.
- Protein concentration increased under high infection pressure, but its relationship with yield loss and disease index was weak, reflecting its compositional nature and dependence on the balance between carbohydrate deposition and nitrogen remobilization rather than on disease DI per se.

Overall, these findings indicate that yellow rust effects on wheat yield and quality are mediated by genotype-specific source–sink adjustment and stress tolerance mechanisms. From a practical perspective, breeding and disease management strategies should therefore prioritize physiological tolerance and source–sink efficiency, rather than relying solely on infection level or yield metrics, to achieve stable grain quality under evolving pathogen races.

## Figures and Tables

**Table 1:** Climatic conditions in 2016, 2021, and 2023 at Rimski Šančevi (Serbia).

Year	T Feb. °C	Prec. Feb. mm	T March °C	Prec. March mm	T April °C	Prec. April mm	T May °C	Prec. May mm
2016	10.5	98.4	7.8	65,6	14.2	74.5	16.9	85
2021	4.9	59.5	6.2	42.8	9.6	55.1	16.0	62.9
2023	3.6	57.2	9.0	25.3	10.5	63.9	17.2	124.8
Average 2006–2023	3.3	44.1	7.4	43.0	12.8	43.5	17.3	91.0

**Table 2:** The most influential factors affecting yield, thousand kernel weight (TKW), and test weight (TW) of 11 commercial winter wheat varieties grown over three years (2016, 2021, and 2023) at the Rimski Šančevi site in Serbia.

Dependent Variable: Yield					
Source	DF	Adj SS	Adj MS	F-Value	*p*-Value
Regression	13	545.447	41.9575	91.10	0.000
DI Yellow rust	1	21.120	21.1198	45.86	0.000
Year	2	101.735	50.8675	110.45	0.000
Variety	10	13.784	1.3784	2.99	0.019
Error	19	8.751	0.4606		
Total	32	554.198			
R^2^ = 98.36%					
Dependent variable: thousand kernel weight (TKW)					
Source	DF	Adj SS	Adj MS	F-Value	*p*-Value
Regression	12	2201.3	183.44	14.85	0.000
Year	2	1864.0	932.01	75.46	0.000
Variety	10	337.2	33.72	2.73	0.027
Error	20	247.0	12.35		
Total	32	2448.3			
R^2^ = 89.91%					
Dependent variable: test weight (TW)					
Source	DF	Adj SS	Adj MS	F-Value	*p*-Value
Regression	13	1336.49	102.807	18.83	0.000
DI Yellow rust	1	36.28	36.275	6.64	0.018
Year	2	48.24	24.118	4.42	0.027
Variety	10	133.39	13.339	2.44	0.045
Error	19	103.73	5.459		
Total	32	1440.22			
R^2^ = 92.80%					

**Table 3:** Spearman’s correlation coefficients between test weight (TW) and thousand kernel weight (TKW) with yield and yellow rust disease index (DI) in 2016 and 2023.

	DI Yellow Rust	Yield
Test weight	r = −0.847, *p* < 0.001	r = 0.934, *p* < 0.001
TKW	r = –0.737, *p* < 0.001	r = 0885, *p* < 0.001

## Data Availability

The original contributions presented in this study are included in the article. Further inquiries can be directed to the corresponding authors.
